# A Novel Technique to Reject Artifact Components for Surface EMG Signals Recorded During Walking With Transcutaneous Spinal Cord Stimulation: A Pilot Study

**DOI:** 10.3389/fnhum.2021.660583

**Published:** 2021-06-03

**Authors:** Minjae Kim, Yaejin Moon, Jasmine Hunt, Kelly A. McKenzie, Adam Horin, Matt McGuire, Keehoon Kim, Levi J. Hargrove, Arun Jayaraman

**Affiliations:** ^1^Shirley Ryan AbilityLab, Chicago, IL, United States; ^2^Feinberg School of Medicine, Northwestern University, Chicago, IL, United States; ^3^Interaction and Robotics Research Center, Korea Institute of Science and Technology (KIST), Seoul, South Korea; ^4^Department of Mechanical Engineering, Pohang University of Science and Technology, Pohang, South Korea

**Keywords:** neurorehabilitation, electrical stimulation, surface electromyography, artifact removal, signal assessment

## Abstract

Transcutaneous spinal cord electrical stimulation (tSCS) is an emerging technology that targets to restore functionally integrated neuromuscular control of gait. The purpose of this study was to demonstrate a novel filtering method, Artifact Component Specific Rejection (ACSR), for removing artifacts induced by tSCS from surface electromyogram (sEMG) data for investigation of muscle response during walking when applying spinal stimulation. Both simulated and real tSCS contaminated sEMG data from six stroke survivors were processed using ACSR and notch filtering, respectively. The performance of the filters was evaluated with data collected in various conditions (e.g., simulated artifacts contaminating sEMG in multiple degrees, various tSCS intensities in five lower-limb muscles of six participants). In the simulation test, after applying the ACSR filter, the contaminated-signal was well matched with the original signal, showing a high correlation (*r* = 0.959) and low amplitude difference (normalized root means square error = 0.266) between them. In the real tSCS contaminated data, the ACSR filter showed superior performance on reducing the artifacts (96% decrease) over the notch filter (25% decrease). These results indicate that ACSR filtering is capable of eliminating artifacts from sEMG collected during tSCS application, improving the precision of quantitative analysis of muscle activity.

## 1. Introduction

Several studies have demonstrated that spinal stimulation has helped restore functionally integrated neuromuscular control of gait in individuals with spinal cord injury (Carhart et al., 2004; Hofstoetter et al., 2015; Minassian et al., 2016; Angeli et al., 2018; Gill et al., 2018; Wagner et al., 2018). Based on these notable results, it has been suggested that transcutaneous spinal cord stimulation (tSCS) can potentially be a simple, safe and noninvasive application in a wide range of neurological diseases with gait disorders. Spinal stimulation operates on the principle that the stimulation exploits spared and silent descending pathways within the spinal circuitry to enable activation of the lower-limb muscles, thus restoring voluntary control of walking (Taccola et al., 2018). Previous investigations primarily highlighted functional recoveries (e.g., restored ability to walk overground, reduced amount of physical support) as the effect of spinal stimulation, but did not investigate detailed changes in neuromuscular control during walking (Angeli et al., 2018; Gill et al., 2018; Wagner et al., 2018). Therefore, it is important to establish sensitive measurement tools to further understand whether recovery or remodeling of neurophysiological factors occur as underlying mechanisms of tSCS effects on functional recoveries.

Currently, one way to test and evaluate the neuromuscular effects of spinal stimulation is with the acquisition of surface electromyograms (sEMG) (Harkema et al., 2011; Grahn et al., 2017; Angeli et al., 2018; Gill et al., 2018; Wagner et al., 2018). One key technical challenge of using sEMG to evaluate stimulation effects is dissociating the net muscle activity from signal artifacts. Specifically, the electrical current from tSCS propagates along skin tissues and contaminates sEMG signals (Mandrile et al., 2003; Qiu et al., 2015). The characteristics of stimulation-induced artifacts depend on the stimulation setting such as stimulation intensity, frequency, and the distance between stimulation location and sEMG electrodes (Qiu et al., 2015). Additionally, there are several intrinsic and extrinsic sources of baseline noise including the amount of fatty tissue between the skin and the muscle tissue, skin-electrode interface, thermal noise, and power line noise (De Luca et al., 2010). Together, these noise sources generate various forms of sEMG artifacts, which might lead to erroneous interpretations of sEMG concerning the neuromuscular effects of tSCS.

Previous tSCS studies filtered the stimulation-induced artifact using reference sEMG electrodes placed over paraspinal muscles to record the artifacts from tSCS (Harkema et al., 2011; Angeli et al., 2018; Gill et al., 2018). However, the frequency characteristics of the signal artifacts can be different between electrodes placed at different muscle locations, so the effectiveness of the performance of this filtering method is uncertain. One study used a notch filter rejecting all signals at a stimulation frequency (Grahn et al., 2017), which might also eliminate the intended sEMG signal originated in muscle activity. Due to these limitations, a more delicate filtering tool that allows precise quantitative analysis of the muscle activity has been needed.

Other electrical stimulation applications (e.g., functional electrical stimulation, direct current brain stimulation) also proposed filtering methods to remove artifacts including signal decomposition methods (e.g., wavelet transform, empirical mode decomposition) (Yochum and Binczak, 2015; Pilkar et al., 2016). These methods are highly effective at removing the artifacts when sophisticated selections are made for filter specifications, such as threshold or mother wavelet. However, determining filter specification is a time-consuming process since there are many factors that must be taken into consideration. Alternatively, hardware-assisted artifact removals have been suggested (Tracey and Krishnamachari, 2006; Wichmann and Devergnas, 2011), but it requires modification of hardware settings and is less flexible than software based approaches.

Recently, our research group developed a novel filter, Artifact Component Specific Rejection (ACSR), that specifically rejects crosstalk for robust gesture recognition (Kim et al., 2020a,b). The development of the ACSR filter was led by the insight that sEMG signals from a specific muscle activation have distinguishable characteristics in frequency domain compared to those from other muscles' activation (i.e., crosstalk). Similarly, we expected that this novel filter could be an effective and time-efficient method to automatically distinguish signal features of muscle activity from those of artifacts originated from diverse sources (e.g., tSCS artifacts, power line noise, intrinsic noise). In this study, we tested whether the ACSR filter could optimize the removal of baseline noise and tSCS artifacts while retaining the maximum amount of intended sEMG signals from simulation data as well as real tSCS contaminated data recorded under diverse tSCS settings and intrinsic and extrinsic conditions.

## 2. Materials and Methods

### 2.1. Participants

Six stroke survivors with diverse demographic characteristics and varied gait impairment levels were recruited for this study (Table 1). Inclusion criteria for all participants included: (1) 18 years of age or older, (2) at least 6-months post-stroke, (3) hemiparesis/hemiplegia after a single stroke, (4) Functional Ambulation Category of two or greater, (5) no presence of severe lower-limb spasticity, (6) no presence of painful musculoskeletal dysfunction, (7) no history of seizures, and (8) no metal implants in the spine or back. Each participant provided informed consent. These procedures were approved by the Northwestern University Institutional Review Board. Prior to the spinal stimulation experiment, self-selected walking speed and the lower extremity motor subscale of the Fugl-Meyer (FMA-LE) assessment (Fugl-Meyer et al., 1975) were measured for each participant to characterize the level of gait impairment. An FMA-LE score of 21 out of 34 was reported to be the optimal cutoff score to differentiate stroke survivors with high or low mobility function (Kwong and Ng, 2019). Additionally, a physical therapist assessed the Functional Ambulation Category score, which distinguishes walking ability on the basis of the amount of physical support required ranging from 0 (unable to walk) to 5 (able to walk independently anywhere) (Mehrholz et al., 2007).

**Table 1:** Participant characteristics.

**Sub #**	**Sex**	**Age (yrs)**	**Time since stroke (yrs)**	**Type of stroke**	**Height (cm)**	**Weight (kg)**	**Paratic side**	**Gait speed (m/s)**	**FMA-LE (Max:34)**	**FAC (Max:5)**	**Stimulation intensity range (mA)**
1	M	67	5	Hem	172.7	77.9	L	0.37	14	2	65–135
2	F	56	2	Isc	162.6	83.3	L	0.81	22	4	30–85
3	M	59	2	Isc	176.8	80.1	R	0.75	23	4	35–105
4	M	61	9	Hem	163.0	70.0	R	0.84	23	4	80–140
5	M	53	6	Isc	175.3	117.9	L	1.00	27	4	45–105
6	M	64	6	Isc	180.0	92.0	L	0.51	18	3	45–90
**AVG**	**–**	**60.0**	**5.0**	**–**	**171.7**	**86.9**	**–**	**0.71**	**21.17**	**3.50**	**–**
**SD**	**–**	**5.1**	**2.7**	**–**	**7.3**	**16.8**	**–**	**0.23**	**4.54**	**0.84**	**–**

*F, Female; M, Male; Isch, Ischemic; Hemo, Hemorrhagic; FMA-LE, Fugl-Meyer Lower Extremity; FAC, Functional Ambulation Category*.

### 2.2. Testing Procedures

**Figure 1:**
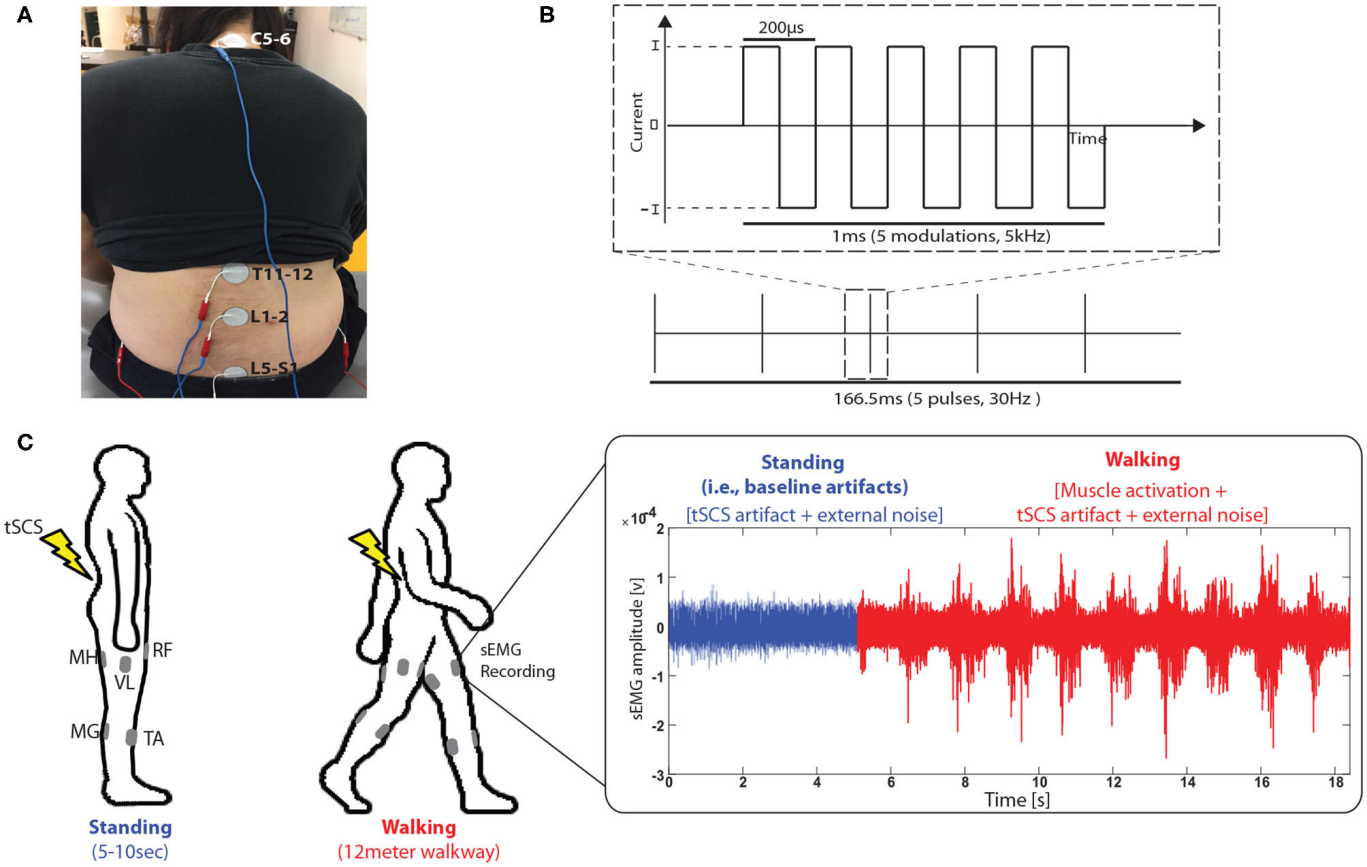
Experimental setup. **(A)** Transcutaneous spinal cord stimulation (tSCS) setup. tSCS was delivered using a surface electrode on the skin between the C5-6, T11-12, L1-2, and L5-S1 spinous process (cathode) and a surface electrode in each anterior crest of the hip bone (anode). **(B)** Schematic representation of the type of current used during tSCS and **(C)** sEMG data collection procedure during walking when tSCS was applied. sEMG signals were recorded from rectus femoris (RF), vastus lateralis (VL), medial hamstring (MH), tibialis anterior (TA) and medial gastrocnemius (MG).

Varying conditions of tSCS were tested for each participant. A custom-built constant current spinal stimulator (BioStim-5, Cosyma, Moscow, Russia) (Grishin et al., 2017) provided tSCS during the assessment. The tSCS was applied at C5-6, T11-12, L1-2, and L5-S1 spinous processes (cathode, Figure 1A), following the tSCS configuration that was used in previous studies for lower-limb rehabilitation (Minassian et al., 2016; Taccola et al., 2018). Ground electrodes were placed on the anterior iliac crests bilaterally (anode). T11 and L1 were stimulated at the subthreshold intensities of 25 , 50 , and 75 % of the resting motor threshold (RMT). Additionally, we added stimulation at L5 and C5, which was held constant at 40 mA. A continuous, biphasic waveform with rectangular 1 ms pulses at a frequency of 30 Hz, with each pulse filled with a modulation frequency of 5 kHz was utilized for all stimulation (Figure 1B). Once a specific stimulation condition was set, the participant remained in a standing position for several seconds for a baseline sEMG recording of each trial (Figure 1C, right). Then, the walking trial began where a participant walked across a 12 m walkway at a self-selected speed. During the testing, Surface EMG (Delsys Trigno, Delsys Inc., Boston, MA) was recorded from five muscles per leg (Figure 1C, left): rectus femoris (RF), vastus lateralis (VL), medial hamstring (MH), tibialis anterior (TA) and medial gastrocnemius (MG). All sEMG data was collected at 2.000 Hz with a bandwidth set to 20–450 Hz (EMGworks Acquisition, Delsys Inc., Boston, MA).

### 2.3. The ACSR Filter Application

#### 2.3.1. Working Principle of the ACSR Filter

sEMG signals represent a summation of signals induced by various sources including tSCS, environmental noise sources, and muscle activations. Importantly, the signals induced by each source have distinguishable characteristics in power spectrum density.

Specifically, signals induced by tSCS, which are referred to as tSCS artifacts in this paper, have specific frequency patterns depending on the combination of modulation frequencies. Environmental noise sources include line interference from power lines, lights, and amplifier circuits. Most environmental noise is considered as white noise (i.e., power at each frequency has similar power at a given frequency bandwidth); power line noise depends on local power-line frequency (50 or 60 Hz). sEMG signals induced by muscle activations are complex and depend on the characteristics of muscles and electrodes. In general, the bandwidth of the sEMG signals induced by muscle activations is considered to be 20–450 Hz.

Therefore, ACSR filter was designed upon the principle that artifact-free sEMG signals can be obtained by first identifying the frequency distribution of the artifacts, then removing those artifact components from artifact-contaminated signals in the frequency domain, and finally reconstructing the artifact-free signals in the time domain.

To reflect this working principle, the algorithm of the ACSR filter was designed to automatically identify the frequency distribution of artifacts (i.e., artifact parameters) from sEMG signals that were recorded with the presence of the artifact sources only with a minimum amount of volitional muscle activation necessary for maintaining standing posture. Although this signal includes some sEMG signals induced by muscle activation for maintaining standing posture, signal amplitude in this portion was considerably smaller (6.7% in average, see Supplementary Materials S2) when compared to signal amplitude recorded during walking. Considering that the signals recorded during standing also include environmental noises, sEMG signals solely induced by muscle activity for maintaining standing posture are presumably minor compared to signals induced by walking. Therefore, we referred to this portion of the signal as artifact-dominant signal, and used this portion as reference signals to identify the frequency distribution of artifacts. Then, the identified magnitudes of the artifacts in the frequency domain are extracted from that of the sEMG recorded during walking under the presence of artifacts.

#### 2.3.2. Filtering Steps

**Figure 2:**
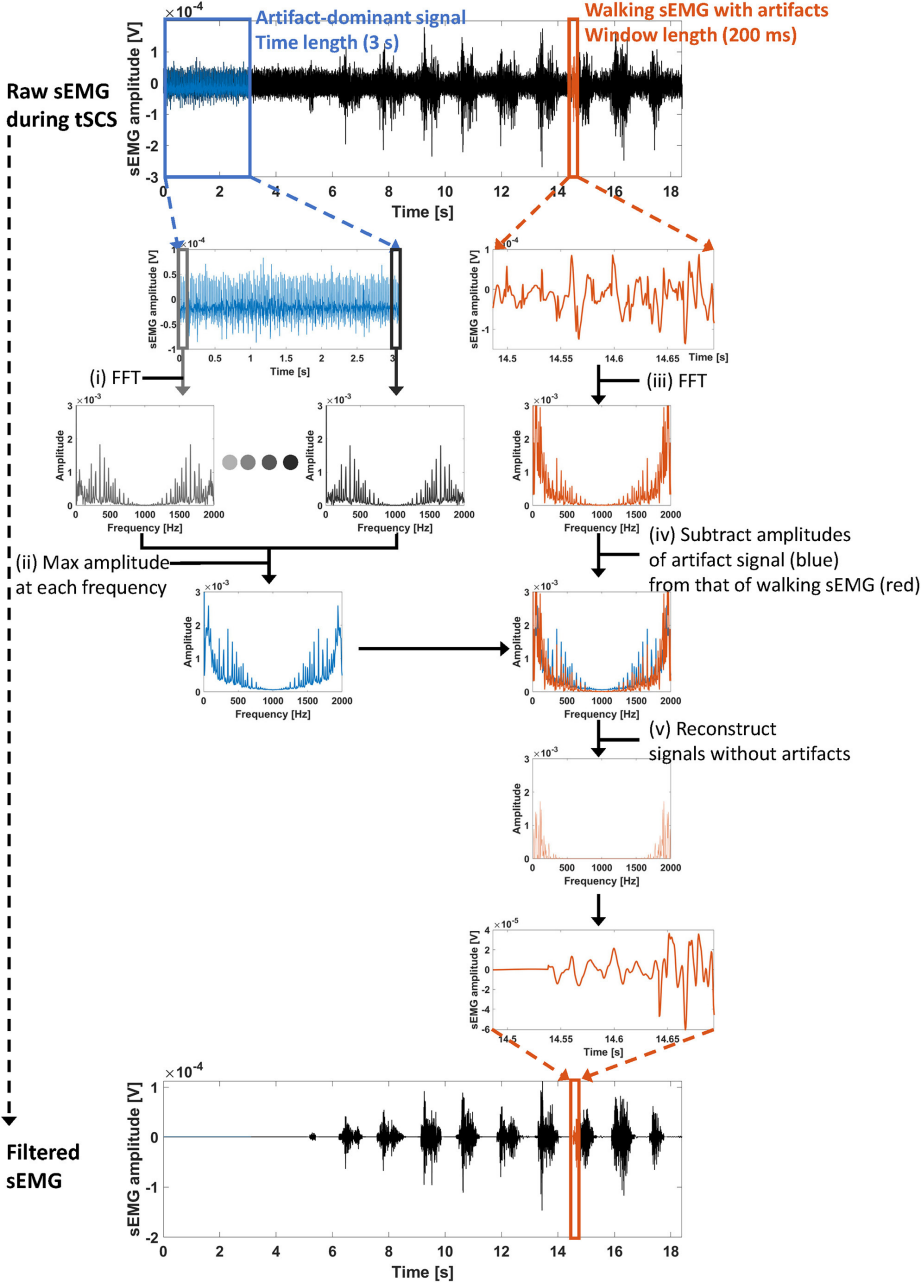
Overall processing scheme of the ACSR filter. **(i)** First, artifact-dominant signals (blue lines, time length = 3 s) were selected to identify artifact parameters. At each segmented window (200 ms), a fast Fourier transform (FFT) was conducted to extract frequency components of the signals within the window. **(ii)** Then, maximum amplitude at each frequency index was obtained. The set of maximum amplitudes at each frequency index indicates the artifact parameters. **(iii)** To identify frequency components of signals recorded during walking (red lines), a FFT was conducted with the signals within segmented windows. **(iv)** Then, the overlapped amplitudes between artifact-dominant signals (blue line) and walking signals (red line) in the frequency domain were subtracted from the amplitudes of the walking signals. The remaining amplitude at each frequency index indicates the frequency of uncontaminated muscle-induced signals. **(v)** Finally, using an inverse FFT, the sEMG signal within the window was reconstructed.

The ACSR filter utilizes a sliding window segmentation to obtain a set of filtered signals. Figure 2 describes the overall filtering scheme. Each step of the filtering process is described below:

(i): Extraction of artifact-dominant signals for filter training

Artifact-dominant signals are measured (Figure 2, blue lines) where sEMG signals were recorded during standing. Since all stimulation was delivered at the subthreshold intensities, there was no stimulation evoked muscle activities. Then, the segmented window and frequency components in each window is extracted using FFT as follows:

(1) $$\begin{array}{l} \left[x_{1},x_{2},\cdot \cdot \cdot ,x_{L}\right]=f_{seg}\left(x_{a}\right)\\ \;y_{l}=\text{fft}\left(x_{l}\right),\;l=1,2,\cdot \cdot \cdot ,L \end{array}$$

where *x*_*a*_ denotes tSCS artifact-dominant signal, and $x_{l}\in R^{N}$ and $y_{l}\in C^{N}$ denote a segmented signal in the window of length *N* and its converted frequency domain signal, respectively. A relationship between the number of windows, *L* and the overlapping window, *t* is represented as follows:

(2) $$\begin{array}{l} L=\left\lfloor \frac{NS-N}{N-t}\right\rfloor +1 \end{array}$$

where *NS* denotes the time length for filter training.

(ii): Identification of artifact parameters

Artifact parameters are extracted by taking the maximum amplitude of each frequency component among the amplitudes obtained in all segmented windows, as follows:

(3) $$\begin{array}{l} Y_{\text{artifact}}^{n}=\underset{1\leq l\leq L}{\max}|y_{l}^{n}| \end{array}$$

where $\left|y_{l}^{n}\right|$ denotes amplitude of the *n*th frequency component of *y*_*l*_, and $Y_{\text{artifact}}^{n}$ denotes *n*th component of the artifact parameters.

(iii): Extraction of a window-segmented signal to apply the ACSR filter

After the identification of the artifact parameters, artifact-filtered signal can be extracted from the signal in window length of N. Here, an arbitrary signal was chosen for demonstration (Figure 2, red lines).

(iv) and (v): Filter application and reconstruction

Then, frequency components of sEMG signals recorded with intended movement are analyzed as follows:

(4) $$\begin{array}{l} \;y=\text{fft}(x)\\ |y^{n^{\prime }}|=\{\begin{array}{l} |y^{n}|-Y_{\text{artifact}}^{n},\text{ if }(|y^{n}|-Y_{\text{artifact}}^{n})\geq 0\\ 0\text{ otherwise} \end{array}\\ \;∠y^{n^{\prime }}=∠y^{n},\;\;\;n=1,\cdots ,N\\ \;x^{\prime }=\text{ifft}(y^{\prime }) \end{array}$$

where *x* and *y* denote the sEMG signal in time domain and frequency domain, respectively, |*y*^*n*^| and ∠*y*^*n*^ denote amplitude and phase of *n*th frequency component, respectively. In conclusion, the filtered signal *x*′ has the same phase as the original signal *x*; the only magnitude for each frequency component is changed.

*x* is generally represented as windows with an overlap of *t*. The artifact-filtered signal, *x*′ is represented as follows:

(5) $$\begin{array}{l} x^{\prime }\left[t+1:N\right]=h\left(x,Y_{\text{artifact}}\right)\left[t+1:N\right] \end{array}$$

where (4) is represented as *h*(·).

#### 2.3.3. Parameter Selection

There are three parameters for the ACSR filter: (1) window length when segmentally analyzing the artifact-dominant signal to extract artifact features (i.e., window length, *N*); (2) size of the overlap between the windows (i.e., overlapping window, *t*), and (3) total time length of artifact-dominant signals that is used for filter training (i.e., time length for training, *NS*). In this paper, window length (*N*), overlapping window (*t*) and time length for training (*NS*) were set as 200 ms, 100 ms, and 3 s, respectively. The selection of the window length (*N*) and the overlapping window (*t*) was based on an empirical ground explored in our previous study (Kim et al., 2020a,b) which observed that these parameters yielded decent filtering performance.

### 2.4. Performance Evaluation

The effectiveness of the ACSR filter was examined on simulated and real-tSCS artifact contaminated sEMG signals from the six participants.

#### 2.4.1. Evaluation With Simulated Artifact

The performance of the ACSR filter was evaluated with an assumption that the filter is capable of restoring sEMG signals contaminated by simulated artifacts to the signals prior to contamination.

The simulated artifact-contaminated signals were generated with linear combination between an original sEMG (i.e., actual sEMG signal collected during walking without tSCS application) and simulated artifacts following actual tSCS specification described in section 2.2. The level of contamination was manipulated by varying ratios between amplitudes of simulated artifacts and that of the original sEMG signals. The simulated artifact-contaminated sEMG signal was generated as follows:

(6) $$\begin{array}{l} X=S+rA \end{array}$$

where *X* denotes simulated artifact-contaminated signals, *S* denotes original sEMG signals, *r* denotes artifact to signal ratio, and *A* denotes the simulated artifact with the same power of the original sEMG signals; power of the signal was represented by the average of the top 100 signal amplitudes of the signal. The artifact-contaminated signals were simulated in ratios from 0.25 to 4 in increments of 0.25.

Then, the ACSR filter was applied to the original sEMG (*S*) and simulated artifact-contaminated signals (*X*), respectively, as follows:

(7) $$\begin{array}{l} S^{\prime }=\text{ACSR}\left(S\right)\\ X^{\prime }=\text{ACSR}\left(X\right) \end{array}$$

where *X*′ and *S*′ denote simulated artifact-contaminated and original sEMG signals, after applying ACSR filter, respectively.

It was assumed that *X*′ and *S*′ would be identical, if the ACSR filter selectively rejects the signal-specific artifact. To measure this conformity between the signals, correlation coefficient (time domain conformity) and normalized root mean squared error (NRMSE, amplitude conformity) between the signals were calculated. The correlation coefficient was calculated using the cross-correlation function in MATLAB 2015a (MathWorks, Inc., Natick, MA, USA). NRMSE was computed as follows:

(8) $$\begin{array}{l} \text{NRMSE}=\frac{\sqrt{{\left(\underset{i}{\sum }X_{i}^{\prime }-S_{i}^{\prime }\right)}^{2}/N_{X^{\prime }}}}{\sigma_{X^{\prime }}} \end{array}$$

where $N_{X^{\prime }}$ denotes the number of samples of *X*′; $\sigma_{X^{\prime }}$ denotes standard deviation of *X*′; and $X_{i}^{\prime }$ and $S_{i}^{\prime }$ denote *i*th sample of artifact-filtered and original-filtered signals, respectively. Higher values of correlation coefficient and lower values of NRMSE indicate superior performance of the filter.

#### 2.4.2. Evaluation With Real tSCS-Contaminated Signals

Since the genuine sEMG signals are unknown once the signal is contaminated by tSCS artifacts, it is impossible to directly evaluate filtering performance with real tSCS-contaminated signals. Alternatively, the performance evaluation of these signals was investigated with an assumption that the amplitude of the baseline noise (i.e., signal noise recorded prior to voluntarily activating muscles) should be minimized to reflect the minimum level of muscle activity.

To compare the filtering performance of ACSR with a conventional notch filter, sEMG signals recorded under various tSCS conditions were filtered separately by notch filter and ACSR filter. For the notch filter, the third-order Butterworth filter was used which band stop frequencies were manually selected by visual inspection of frequency-domain signals: 62, 94, 125, 156, 188, 219, 250, 282, 313, 345, 376, 407, 439, 470, 501, 533, 564, 595, 627, 658, 690, 721, 752, 784, 815, 846, 878, and 909 Hz. Baseline noise amplitudes were computed by RMS envelopes of the raw, notch-filtered, and ACSR-filtered signals. Lower baseline noise amplitudes were considered to indicate better performance of the filter.

Statistical analysis: Additional statistical analyses were conducted to compare the performance of removing tSCS artifacts (SPSS 26.0, IBM, Inc., Chicago, IL). We compare the baseline noise amplitude among different filtering methods (raw signal, notch-filtered signal, and ACSR-filtered signal) and recording conditions (muscles and tSCS intensity) as factors to assess their influence on the effect of the artifact removal. Two-factor split-plot Analysis of Variance (ANOVA) was computed with filtering methods as a main effect and muscles where sEMG signals were recorded (RF, VL, MH, TA, and MG) as a subplot effect. Similarly, two-factor split-plot ANOVA was computed with filtering methods as a main effect and tSCS intensities (no stimulation, 25 % RMT, 50 % RMT, and 75 % RMT) as a subplot effect. When a significant effect was observed, *post-hoc* paired comparisons were conducted with Fisher's LSD test. All tests were performed as a two-sided test. *P*-values equal to or less than 0.05 were considered statistically significant.

### 2.5. Evaluation on Influence of Filter Parameters on the ACSR Filter Performance

Although the filter parameters were selected based on the empirical evidence in this study (see section 2.3.3), the influence of the three filter parameters (window length, *N*; overlapping window, *t*; time length for training, *NS*) on the ACSR filter performance was further evaluated in two ways: (1) with a real tSCS-contaminated signal, and (2) with simulated signals. First, we evaluated each parameter's influence on the filter performance with an exemplary signal that was contaminated by real tSCS artifacts (subject 3, VL muscle, tSCS intensity: 75% RMT). For this analysis, we systematically adjusted the value of one parameter at a time, while keeping a constant value for the other two parameters. Specifically, to examine the influence of the window length (*N*) on the filter performance, the window length was changed from 100 ms to 800 ms in increments of 50 ms, while keeping the other two parameters constant (*t*= 100 ms, *NS*= 3 s). Similarly, the overlapping window (*t*) was changed from 0 ms to 199 ms in increments of 1 ms, while unchanging the other two parameters (*N*= 200 ms, *NS*= 3 s). Lastly, time length for training (*NS*) was explored from 0.5 s to 6 s in increments of 0.5 s, while keeping the other two parameters constant (*N*= 200 ms, *t*= 100 ms).

Additionally, we performed a similar evaluation with simulated signals. For simplicity of the manuscript, the detailed methods and results with the simulated signals were described in Supplementary Materials S1.

### 2.6. Code Availability

Matlab script implementing the ACSR filter is available online from https://github.com/mjkim0927/kim-frontiers-2021.

## 3. Results

### 3.1. Performance Evaluation Using Simulated Artifact-Contaminated Signal

**Figure 3:**
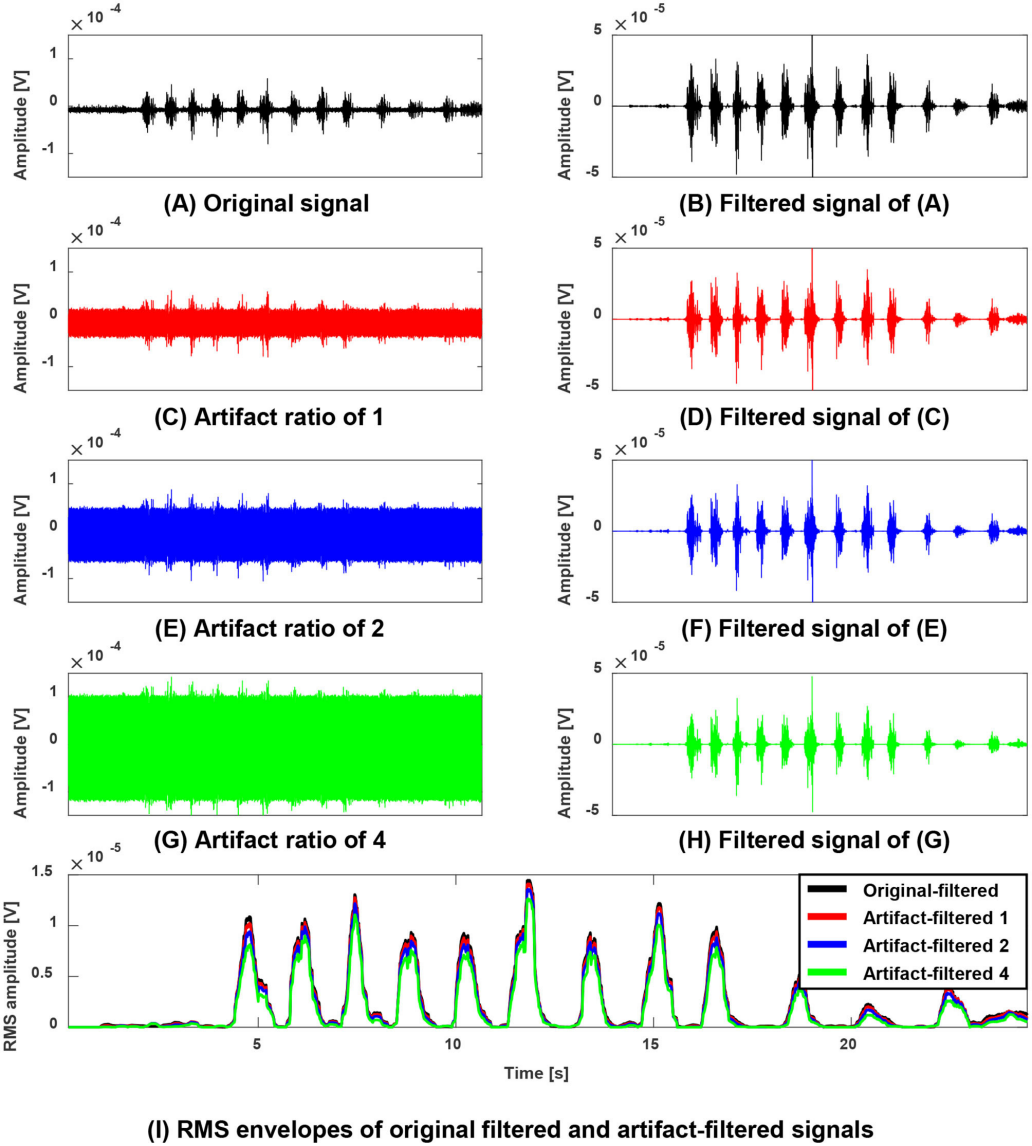
Performance evaluation of the ACSR filter using simulated artifact-contaminated signals **(A,B)** an exemplary original sEMG signal (paretic side RF of subject 6) recorded during walking without tSCS **(A)** and its filtered signal **(B)**. **(C,E,G)** A contaminated sEMG signal generated with linear combination of the original signal and simulated artifacts with artifact-to-signal ratio of 1 **(C)**, ratio of 2 **(E)** and ratio of 4 **(G)**. Their corresponding filtered signals were displayed on the right column **(D,F,H)**. **(I)** Overlaid Root Mean Squared (RMS) enveloped signals of the filtered signals. Note that filtered original signal (black line) and all filtered contaminated signals (red, blue, green lines) are generally well matching with respect to timing and amplitude. This result indicates that the proposed filter selectively rejected the simulated artifacts without degrading or distorting the muscle-induced signals.

Figure 3 shows an exemplary outcome of ACSR filter by comparing the original-signal (paretic side RF of subject 6, Figure 3B) with simulated artifact-contaminated signals (Figures 3D,F,H), after applying the filter to each signal. In this example, the ACSR filter recovered the contaminated signals to be matched with the original signal with respect to timing (correlation coefficients *r* = 0.949 to 0.992) and amplitude (NRMSE = 0.130 to 0.349). The result reflects that the algorithm was able to extract the sEMG signal induced by muscle activity with minimal loss of data in all signals contaminated with various artifact-to-signal ratios.

**Figure 4:**
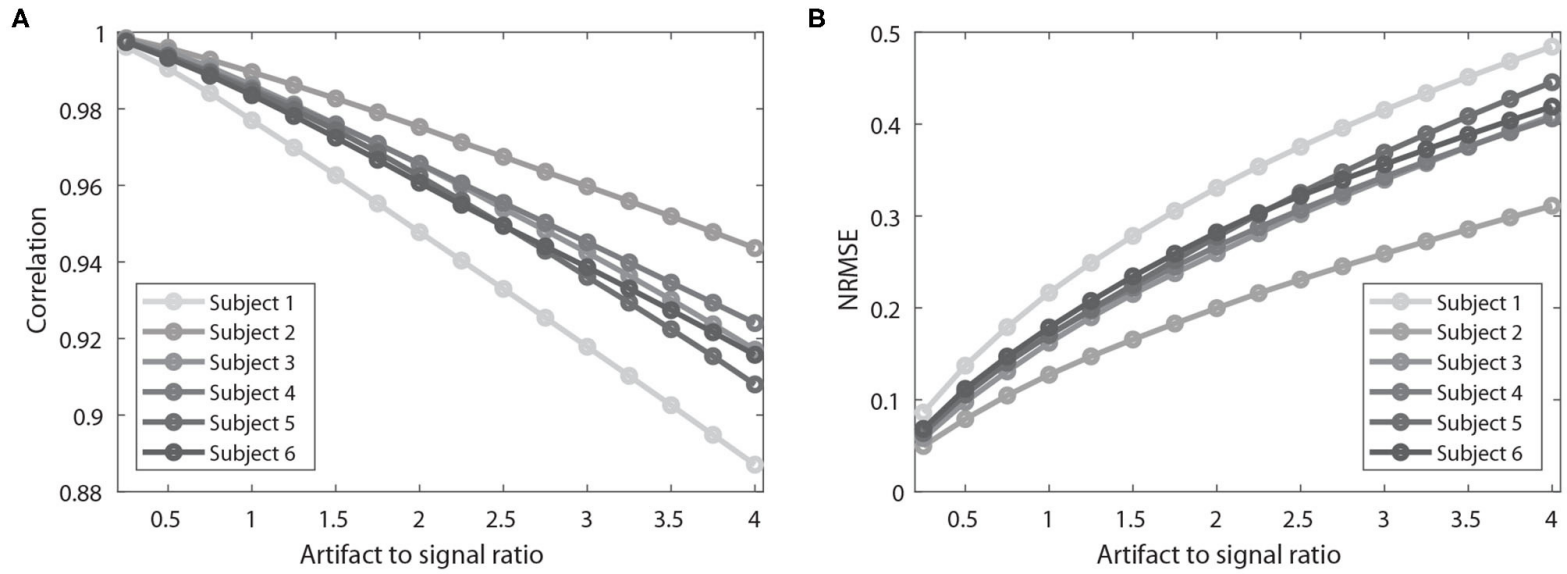
Results of performance evaluation of ACSR filter using simulated artifact-contaminated signals of six participants. **(A)** Results of correlation coefficients between an original signal and simulation artifact-contaminated signal after applying the filter. Higher correlation coefficient indicates better filtering performance. **(B)** Results of normalized root-mean-square error (NRMSE) between the signals. Lower NRMSE indicates better filtering performance. As the artifact to signal ratio increases, there is a decrease in correlation coefficient and increase in NRMSE. This result indicates that the performance of ACSR filter is influenced by the severity of contamination level.

The same analysis was applied to all six participants. Overall, the filtered signal matched well with the original signal (i.e., signal before being contaminated with simulated artifacts) with respect to the timing and amplitude reflected by correlation coefficients (AVG = 0.959) and NRMSE (AVG = 0.266). Notably, the performance of the ACSR filter decreased with increased degree of contamination (Figure 4). In all six participants, as artifact-to-signal ratio increased, conformity of timing and amplitude between the filtered-contaminated signals and filtered-original signal was decreased, reflected by a gradual decrease in correlation coefficient and increase in NRMSE. However, it should be noted that even at an artifact-to-signal ratio of 4, the correlation coefficient ranged between 0.887 and 0.916 and NRMSE ranged between 0.419 and 0.484, indicating a decent level of agreement between filtered-contaminated and filtered-original signals.

### 3.2. Performance Evaluation Using Real tSCS Contaminated Signals

#### 3.2.1. Reducing Baseline Noise of sEMG Recorded From Various Muscles

**Figure 5:**
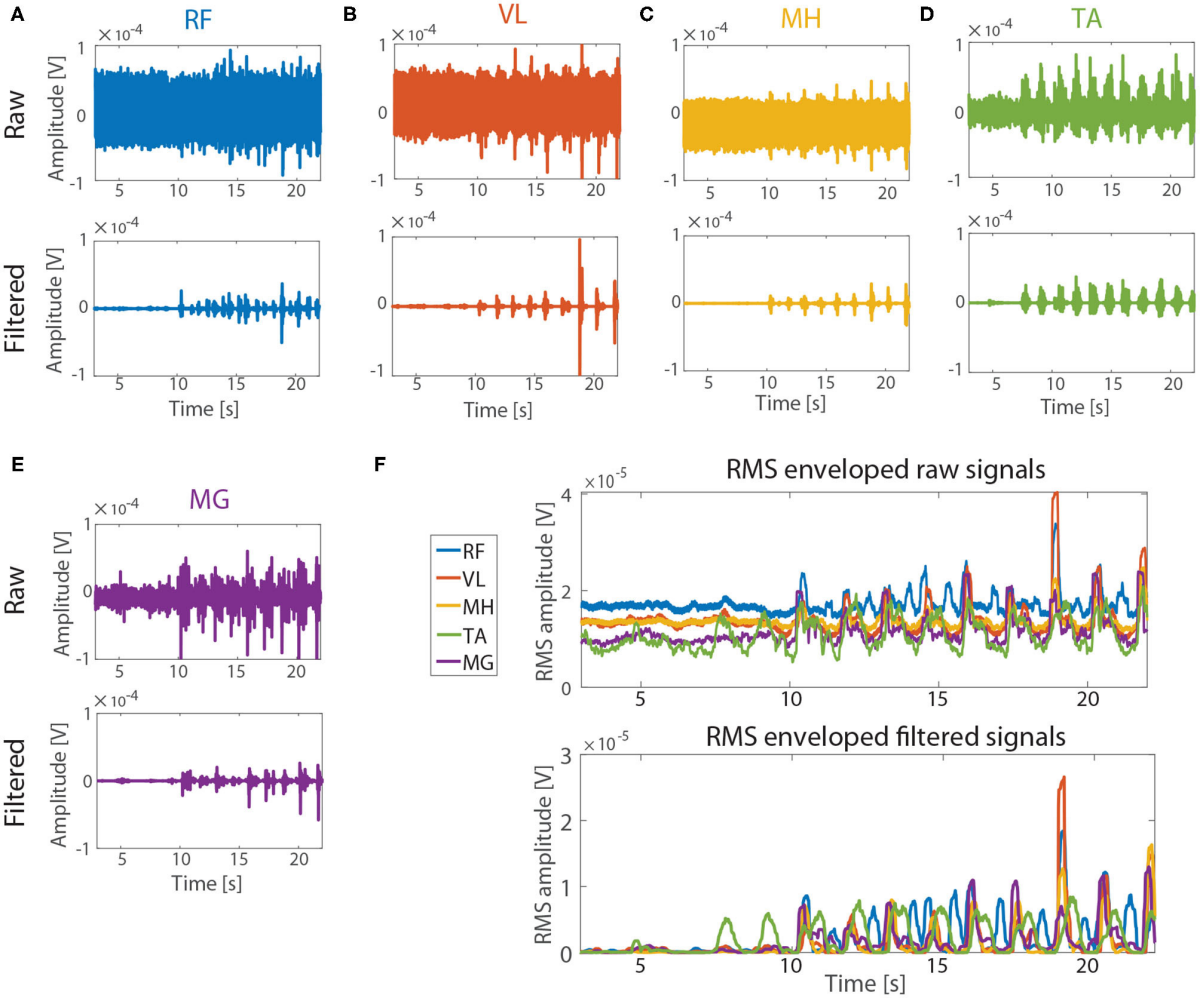
Performance evaluation with real tSCS contaminated sEMG signals. The signals from subject 2 (tSCS intensity: 75 % RMT) were represented as exemplary signals. Raw (upper figures) and filtered (lower figures) signals from RF, VL, MH, TA, and MG are represented in **(A–E)**, respectively. Note that the amplitude of the baseline noise (3–7 s) varied between the muscles in raw signals. **(F)** RMS envelops of the signals before (upper figure) and after (lower figure) applying the ACSR filter. The amplitude of the baseline noise reduced close to 0 V for all five muscles while the muscle activation patterns were preserved.

Figure 5 describes exemplary results (subject2, tSCS intensity: 75% RMT) of reduction of the baseline noise of the real tSCS contaminated sEMG signals of the five muscles (RF, VL, MH, MG, and TA) after applying ACSR filter. The raw data (upper figures, Figures 5A–E) showed that baseline noise (signals between 3 and 7 s) existed and the amplitudes of the noise were significantly different among the muscles. After applying the ACSR filter (lower figures, Figures 5A–E), the baseline noise was reduced.

**Figure 6:**
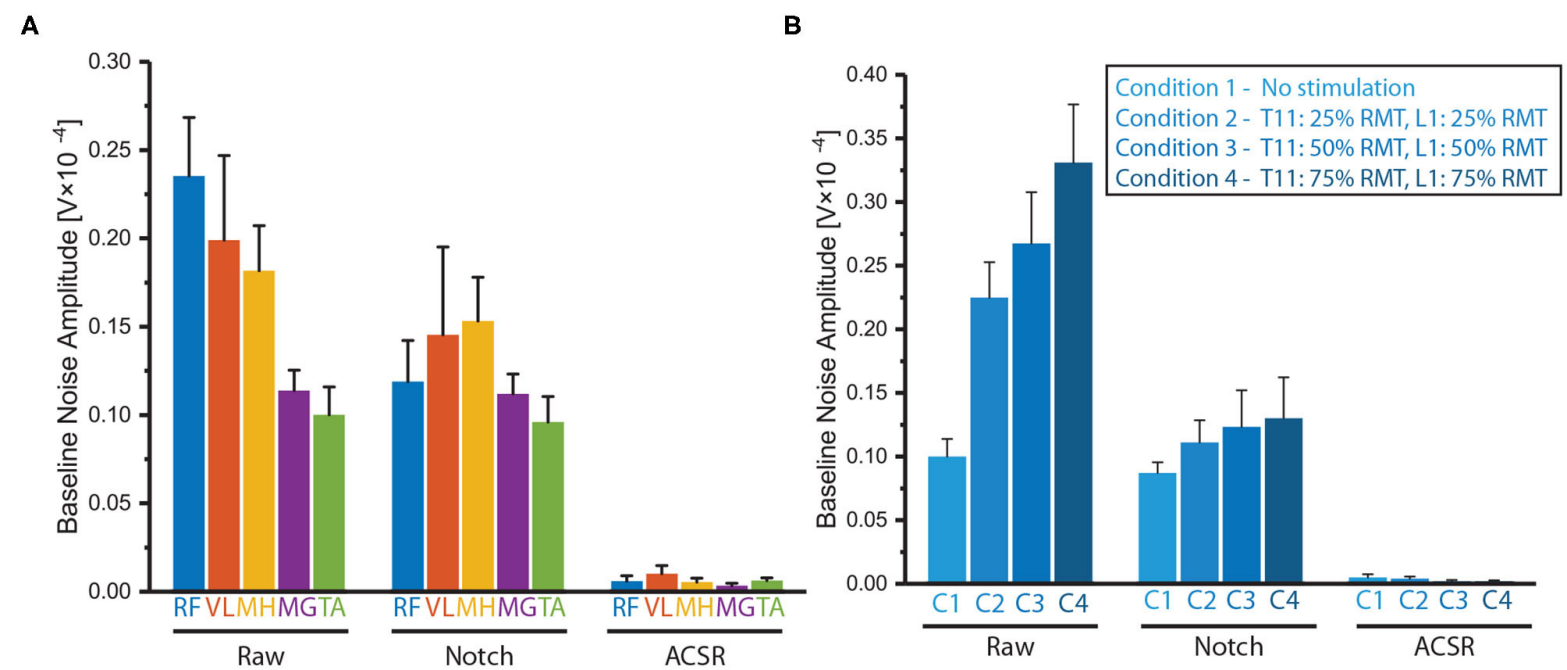
Comparison of baseline noises filtered by notch filter or ACSR. Comparison of baseline noises recorded **(A)** from five muscles (RF, VL MH, MG and TA) and **(B)** from RF muscles in four different tSCS intensities. Overall, the ACSR filter reduced the baseline noise greater than the notch filter. In addition, in the raw data, the baseline noises were significantly greater in the muscles at the thigh (RF, VL, MH) compared to those at the shank (MG, TA) and also when higher tSCS intensities were applied. These differences became insignificant after applying either notch or ACSR filters. Data presented as Mean ± Standard Error.

The same analysis was applied to the contaminated sEMG signals (i.e., raw data) of the paretic side of five muscles of the six participants, while applying the notch filter and ACSR separately (Figure 6A). The statistical results showed that there was significant difference in the baseline noise among the filtering methods (*F*_(2,60)_ = 123.15, *p* < 0.001). As expected, the baseline signal noises were significantly reduced after applying either the notch filter (*p* < 0.001) or ACSR filter (*p* < 0.001). Importantly, the amount of reduction was greater for the ACSR (AVG = 97% reduction) compared to the notch filter (AVG = 25% reduction). Specifically, the ACSR filter significantly reduced baseline noise for all muscles (*p*′*s* < 0.050). On the other hand, the notch filter significantly reduced the baseline noise only for RF muscles (*p* = 0.003), while it did not for other muscles (*p*′*s* > 0.050).

Another interesting observation was that there was significant interaction effect between filtering methods and muscles in the baseline noise (*F*_(8,60)_ = 3.54, *p* = 0.002). Specifically, a *post-hoc* analysis revealed that in the raw data, muscles on the thigh (RF, VL, MH) had significantly greater noise amplitudes compared to those of the muscles on the shank (MG and TA) (*p*′*s* < 0.050). However, the differences of the baseline noise between the muscles became insignificant after applying either the notch filter (*p* = 0.592) or ACSR filter (*p* = 0.549).

#### 3.2.2. Reducing Baseline Noise of sEMG Recorded With Various tSCS Intensities

The notch and ACSR filters were applied separately on the contaminated sEMG of RF muscle on the paretic-side recorded with various tSCS intensities (i.e., no stimulation, 25 % RMT, 50 % RMT, 75 % RMT) (Figure 6B).

There was significant difference in the baseline noise among the filtering methods (*F*_(2,45)_ = 286.60, *p* < 0.001) showing both the notch and ACSR filters significantly reduced baseline noise of the raw data (*p*′*s* < 0.001). However, the amount of reduction was greater for the ACSR filter (AVG = 99% reduction) compared to that of the Notch filter (AVG = 51% reduction).

Additionally, there was significant interaction effect between filtering methods and muscles in the baseline noise (*F*_(6,45)_ = 15.12, *p* < 0.001). Specifically, in the raw data, the baseline noise significantly increased as higher tSCS intensities were applied (*p* = 0.001). However, these differences of baseline noise among the different intensity conditions became insignificant after applying either the notch filter (*p* = 0.587) or the ACSR filter (*p* = 0.423).

#### 3.2.3. Artifact-to-Signal Ratio of Real tSCS-Contaminated Signals

The artifact-to-signal ratio in real tSCS contaminated data sets was estimated indirectly using ACSR methods (see Supplementary Materials S3). The median ratio between the artifact and muscle activation signal was 1.464 (IQR = 0.573−3.425) in the real tSCS-contaminated signals.

### 3.3. Influence of Filter Parameters on Performance

**Figure 7:**
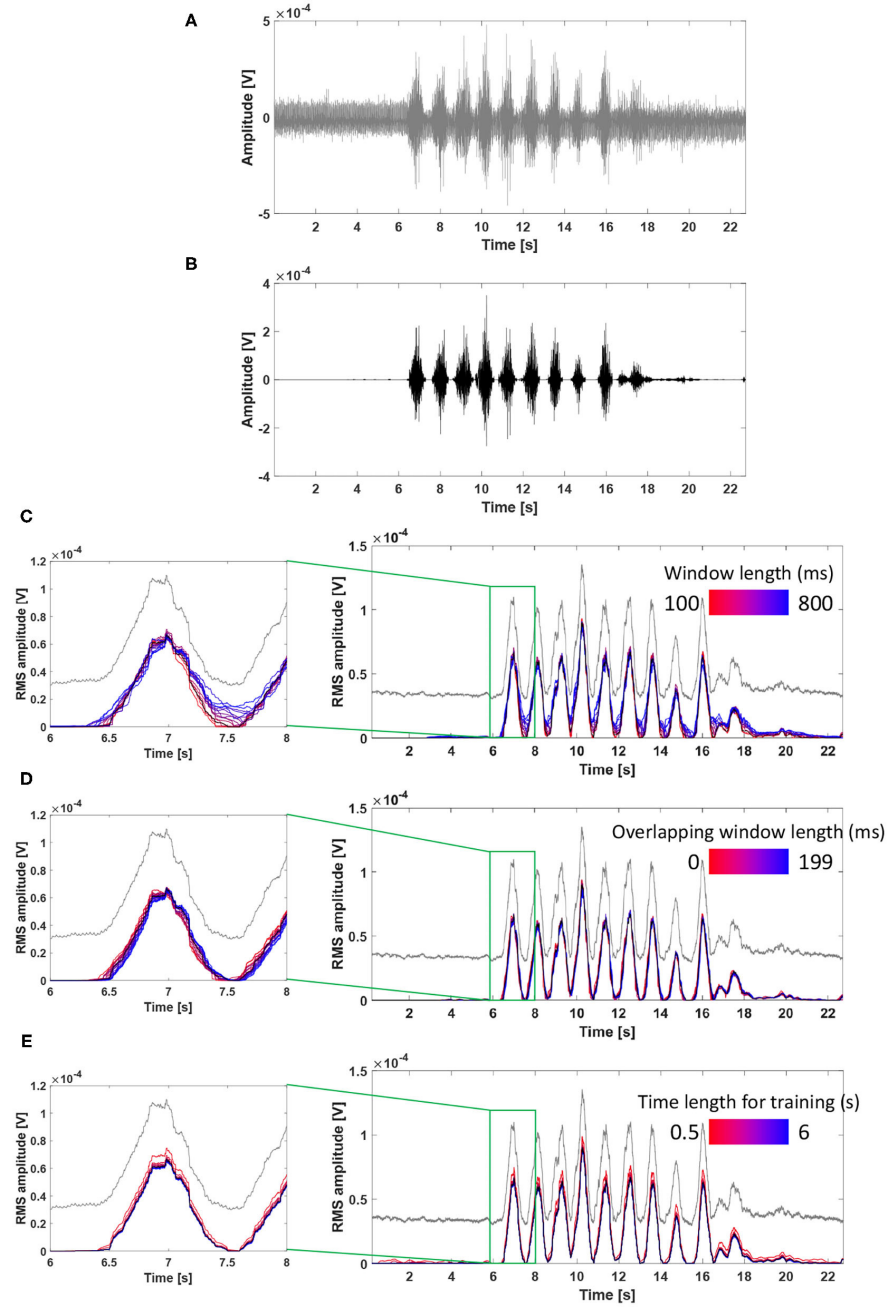
Influence of filter parameters on ACSR filter performance using an exemplary real tSCS-artifact contaminated signal (subject 3, VL, tSCS intensity of 75% RMT). **(A)** The raw signal, **(B)** filtered signal using parameters as the window length of 200 ms, the overlapping window of 100 ms, and the time length of 3 second, **(C)** Influence of window length on the filter performance, **(D)** Influence of overlapping window length on the filter performance. **(E)** Influence of time length for training on the filter performance. RMS envelopes of the raw and filtered signals were shown in gray and black lines from **(C–E)**.

Figure 7 describes the influence of each parameter on performance using an exemplary real tSCS-artifact contaminated signal (subject 3, VL, tSCS intensity of 75% RMT). Figures 7A,B show the raw signal and filtered signal using the parameters as the window length (*N*) of 200 ms, the overlapping window (*t*) of 100 ms, and the time length (*NS*) of 3 s.

Figure 7C describes the effect of change in the window length (*N*) on the filter performance. As the window length increased (from red to blue line), the filtered signals showed higher RMS amplitudes at the phase of muscle deactivation (e.g., signals at 7.5 s) indicating that a greater amount of signal remained unfiltered. This result implies that longer window lengths resulted in a higher frequency resolution, and the complicated frequency distribution of artifacts is likely to be overfitted to the training signals with non-stationary artifacts.

Figure 7D describes the effect of the overlapping window (*t*) on the filter performance. While there were minimal differences among the filtered signals, as size of the overlapping window increased (from red to blue line), the graphs slightly shifted toward the right side (i.e., temporal delay). This result indicates that an elongated overlapping window setting might cause temporal offset of the filtered signal.

Figure 7E describes the effect of change in the time length for training (*NS*). All filtered signals had a similar shape and amplitude, except for the signal filtered with a time length of training of 0.5 s (brightest red line), which showed higher RMS amplitude compared to the other signals. In this exemplary signal, the filter performed similarly when the time length of training was set between 1 and 6 s. In general, a longer time length for training allows to detect more variation of artifacts in the reference signal, which might lead to improved filter performance. However, it should also be considered that extending the time length of training might increase the risk of rejecting muscle-induced signals because a subject might unintentionally contract muscles during this period.

## 4. Discussion

The present study applied the ACSR (i.e., artifact component specific rejection) filter to remove tSCS artifacts from the contaminated sEMGs of stroke survivors data. The ACSR filter was designed to automatically identify components of artifacts in the artifact-dominant-signal (i.e., signals with miminum voluntary activation) and eliminate the identified components from the sEMG signal collected during walking. We evaluated the performance of the ACSR filter with both simulated and actual tSCS artifact-contaminated data and observed that the filter is capable of removing these artifacts.

### 4.1. Performance Evaluation With Simulated Signals Contaminated With Various Artifact-to-Signal Ratios

The performance of the ACSR filter was tested with simulated artifact-contaminated sEMG, which was generated with a linear combination of the sEMG without tSCS (i.e., original signal) and simulated artifacts contaminating sEMG in multiple degrees. Overall, the filtered artifact-contaminated signal matched well with the original signal with respect to the timing and amplitude. However, the performance of the ACSR filter depended on the severity of signal contamination, showing better performance with lower amplitude of simulated-artifacts (i.e., lower artifact-to-signal ratio). In the real tSCS contaminated data sets, the artifact to signal ratio ranged from 0.573 to 3.425. The simulated signals showed that the ACSR filter performed well in this level of contamination, supporting the use of the ACSR filter in lower-limb sEMG during walking when tSCS is applied.

### 4.2. Performance Comparison Between a Previous Filtering Method and ACSR Filter

We compared the performance of the ACSR filters with that of a previous method (i.e., notch filter) with the real tSCS contaminated data by examining the amount of reduction of the baseline noise after applying each filter. The results demonstrated superior performance of the ACSR filter (reduced the baseline noise by 96 %) over the notch filter (reduced it by 25 %). A known issue of the notch filter is that it requires prior information about the stimulation conditions (e.g., frequency) to determine appropriate filter parameters (Qiu et al., 2015). Additionally, the notch filter rejects all signals at a target frequency while frequency components between muscle-induced and artifact-induced sEMG signals are overlapping (Qiu et al., 2015). In contrast, the ACSR filter does not have these limitations because it automatically identifies artifact characteristics and does not need prior knowledge about stimulation conditions. Also, the filter only subtracts magnitude of the signal originated from artifacts while keeping those originated from muscle activity.

Additionally, we observed that the characteristics of baseline noise was significantly influenced by sEMG recording conditions. Specifically, in the raw data, higher baseline noise amplitude was observed in muscles located closer to the tSCS sites (i.e., higher noise at thigh muscles than at shank muscles), and as higher tSCS intensities were applied. Previous spinal stimulation studies used reference sEMG electrodes placed over paraspinal muscles to record tSCS induced artifacts and filtered the recorded artifacts from the lower-limb sEMG (Harkema et al., 2011; Angeli et al., 2018; Gill et al., 2018). However, our results indicate that this previous method might not optimally perform since artifacts recorded at the paraspinal muscles are likely to have different characteristics than those recorded at the lower-limb sEMG. On the contrary, the ACSR filter was designed to identify components of artifacts and eliminate the identified components within the same sEMG data set, minimizing condition differences between the reference and outcome signals. This observation supports that the ACSR filter could allow for precise quantitative comparison of the muscle activity recorded in various conditions.

### 4.3. Potential Application

Our approach shows the potential to evaluate sEMG signals induced by neuromuscular activation during tSCS application, which can provide important information about the effects of tSCS. The effects enabling motor control when delivering tSCS and functional improvements are not fully understood due to the lack of a sensitive tool to dissociate the stimulation artifacts from the underlying muscle activity. Consequently, the effect of tSCS on functional performance has been primarily examined by mechanical output (e.g., gait parameters, joint kinematics) or clinical measures (Gerasimenko et al., 2015; Angeli et al., 2018). While these scales showed functional recovery where participants accomplished walking patterns typically used by nondisabled individuals, detailed evaluation on muscle activation would further inform whether the functional recovery was achieved through neuromuscular recovery or adaptive/substitutive compensatory strategies (e.g., increase agonist/antagonist coactivation, altered muscle activation timing). Therefore, the development of this artifact-filtering algorithm for tSCS may provide more insight into the impact of tSCS performance at a physiological level and help to apply these findings clinically.

Additionally, it is possible to apply this software algorithm within the sEMG acquisition and/or processing platforms, so that this software could instantaneously subtract the artifacts in the sEMG data, without modifying the data acquisition hardware. When applying the ACSR filter for such an online processor, the filter parameters should be carefully re-determined to minimize time delay on recognition of muscle activation (e.g., minimizing the size of the overlapping window).

### 4.4. Limitations and Future Directions

There are a few limitations to be noted. First, the ACSR filter was designed with the assumption that the artifact characteristics would be constant throughout a single data set. However, in a practical situation, artifacts may be continuously changed due to electrode displacement during recording, and interference of nearby electrical equipment. In these situations, pre-trained filtering parameters cannot respond to the changed artifact condition.

The second limitation is that the reference signals (i.e., artifact-dominant signal), in which the artifact component is trained, include stimulation artifacts as well as sEMG activity induced by maintaining standing posture. However, as stated in the method section (section 2.3.1, Supplementary Materials S2), the signal amplitude solely induced by standing posture was substantially smaller compared to the signal amplitude induced by walking. Therefore, we believe that the benefit for applying the filter to analyze walking data exceeds the risk of losing a minimal amount of sEMG signals activated to maintain a standing posture. However, caution is needed when applying the filter, since this method is only validated for examining sEMG activity during walking, and might not be adequate when assessing other activities such as standing.

Another limitation is the small sample size and scarce diversity of gait impairment levels since it was a pilot study to determine feasibility of implementing the ACSR filter when applying tSCS. All six participants included in this study had mild to moderate gait impairment, classified as community ambulatory (Functional Ambulation Category ≥2). Also, based on the FMA-LE score, two out of six participants (Subject 1&6) had low mobility function (FMA-LEM <21), while others had relatively high mobility function. Given that the filter performance gets worse as the ratio between artifact and muscle-induced signal increases, it is possible that the filter does not work properly for a subject with severe weakness of muscle activation. Additionally, while the current study only involved stroke survivors, the performance of the filter could be more associated with the degree of walking deficits than the type of pathology. Therefore, to expand on this pilot study, a future study is warranted to include participants with a broader scope of gait impairments (mild, moderate and severe) as well as healthy controls to allow for comparison of filter performance between the groups. Lastly, it should be noted that the artifact amplitude increased as distance between the sEMG electrode location and stimulation site gets closer (Figure 6A). Therefore, while the current study evaluated muscles on the thigh and shank only, it would be interesting to evaluate the performance of the filter closer to the stimulation area, such as the gluteal muscles.

## 5. Conclusion

We observed that the ACSR filter is capable of eliminating artifacts from sEMG collected during tSCS application in stroke survivors. The ACSR approach demonstrated decent accuracy and robustness to recover sEMG at different levels of contamination. Additionally, the ACSR filter showed superior performance on reducing the baseline noise over the conventional notch filter. Overall, the ACSR filter provides an effective, time-efficient, and easy-to-implement approach to evaluate sEMG signals during tSCS. Therefore, this approach can allow precise quantitative analysis of the muscle activity recorded under diverse tSCS conditions.

## Data Availability Statement

The original contributions presented in the study are included in the article/Supplementary Material, further inquiries can be directed to the corresponding author/s.

## Ethics Statement

The studies involving human participants were reviewed and approved by Northwestern University IRB. The patients/participants provided their written informed consent to participate in this study.

## Author Contributions

MK and YM contributed to the conception and design of the study and wrote the first draft of the manuscript. YM, JH, and KM performed data collection. KM recruited subjects. MK, YM, and JH performed the data analysis. AH and MM reviewed and revised the manuscript. MK and KK developed the method. AJ, KK, and LH acquired funding. AJ supervised and administered the project. All authors contributed to manuscript revision, read, and approved the submitted version.

## Conflict of Interest

The authors declare that the research was conducted in the absence of any commercial or financial relationships that could be construed as a potential conflict of interest.
