# Supplementary material for: A Novel Technique to Reject Artifact Components for Surface EMG Signals Recorded During Walking With Transcutaneous Spinal Cord Stimulation: A Pilot Study
Source: Front Hum Neurosci. 2021 Jun 3;15:660583. doi: 10.3389/fnhum.2021.660583 (PMC8209256; doi:10.3389/fnhum.2021.660583)
Supplement: Supplementary file 1 [file Data_Sheet_1.pdf]

Supplementary Material

S1 INFLUENCE OF FILTER PARAMETERS ON THE ACSR FILTER PERFORMANCE

S1.1 Methods to evaluate filter performance

To evaluate the influence of the ACSR filter parameters for removing artifacts, exemplary signals were generated for both the original and the artifact-contaminated signals using a combination of sine waves. It was assumed that when proper filter parameters are selected, the filter is capable of restoring artifact-contaminated signals to the original signals.

For the generation of the simulated signals, each signal at a time, t, is represented as follows:

$$\begin{aligned} v &= f(h, a_0, t_{start}, t_1, t_2, t_{stop}) \\ &= g(a_0, t_{start}, t_1, t_2, t_{stop}) * \sin(2\pi h t) \end{aligned} \tag{S1}$$

where $g(\cdot)$ is represented as follows:

$$g(a_0, t_{start}, t_1, t_2, t_{stop}) = \begin{cases} a_0(\frac{t-t_{start}}{t_1-t_{start}}), & \text{if } (t_{start} \leq t < t_1) \\ a_0, & \text{if } (t_1 \leq t \leq t_2) \\ a_0(1-\frac{t-t_2}{t_{stop}-t_2}), & \text{if } (t_2 < t \leq t_{stop}) \\ 0, & \text{otherwise} \end{cases} \tag{S2}$$

Original signals (i.e., signals without artifacts) with two activation periods, were created as follows:

$$\begin{aligned} v_1 &= f(25, 1, 1, 1.8, 2.9, 3) + f(40, 2, 1, 1.8, 2.9, 3) + f(135, 1.5, 1, 1.8, 2.9, 3) \\ v_2 &= f(250, 3, 4, 4.1, 5.8, 6) + f(700, 1, 4, 4.1, 5.8, 6) + f(75, 2, 4, 4.1, 5.8, 6) \end{aligned} \tag{S3}$$

where all signals were sampled at 2000 Hz Hz. v_1 represents signal activation from 1–3 seconds, and v_2 represents signal activation from 4–6 seconds shown in figure S1A.

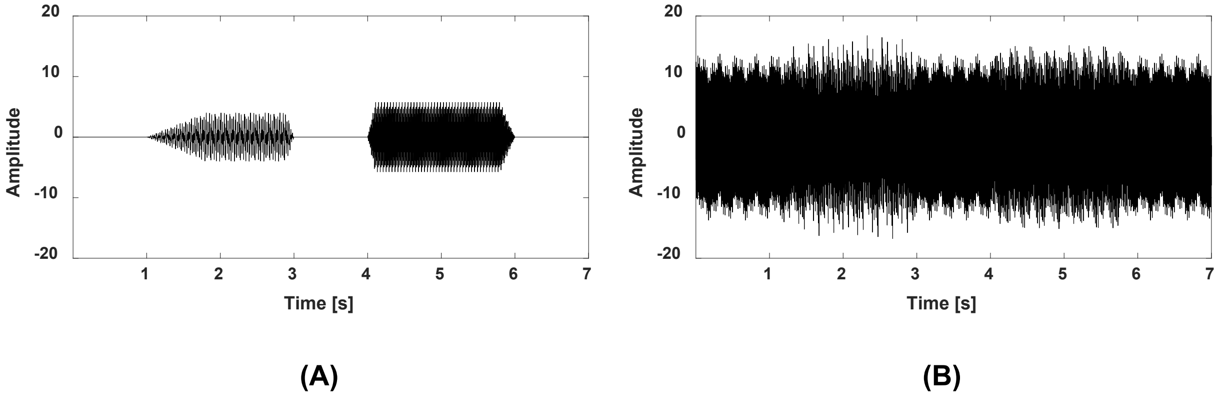

Figure S1. Simulated signals used for performance evaluation; **(A)** original and **(B)** artifact-contaminated signals.

To simulate artifacts, arbitrary signals were created as follows:

$$v_{art} = f(4,1,0,0,7,7) + f(120,4,0,0,7,7) + f(450,5,0,0,7,7) + f(900,5,0,0,7,7) \quad \text{(S4)}$$

Then, artifact-contaminated signals were generated with a summation of the original signals and artifacts (figure S1B).

To evaluate the performance of the filter, Root Mean Square Error (RMSE) between the original and artifact-contaminated signals after applying the ACSR filter (i.e., filtered signal) were computed as follows:

$$RMSE = \sqrt{\frac{\sum_{i=1}^{n}(x_i^t - x_i^f)^2}{n}} \quad \text{(S5)}$$

where x^t denotes an original signal, x^f denotes a filtered signal, and n denotes the number of signal points.

S1.2 Default values of the filter parameters

There are three parameters for the ACSR filter: 1) the window length, 2) the size of the overlap between the windows (i.e., overlapping window), 3) the total time length of artifact-dominant signals that are used for filter training (i.e., time length for training). Default values for these parameters were set as described in Table S1.

Parameter	Initial value
Window length	0.2 s
Window overlap	0.1 s
Time length for training	1 s

Table S1. Default values of the filter parameters.

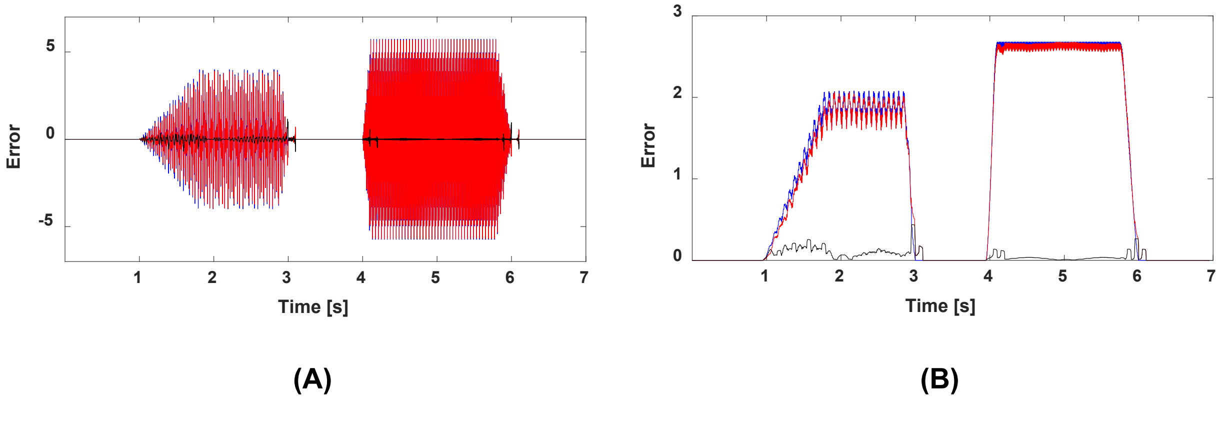

Figure S2. ACSR filter application using the default parameter values. **(A)** a high similarity between the original (blue line) and the filtered signal (red line) was obtained; the difference between the signals was represented with a black line. **(B)** RMS envelopes of **(A)**.

Figure S2 shows the comparison between the original and the filtered signals using the default filter parameters. RMSE between the original and filtered signals was 0.0818.

These default parameters were used to evaluate the influence of each parameter on filter performance. Specifically, we adjusted the value of one parameter at a time, while keeping the default values for the other two parameters.

S1.3 Influence of window length on filter performance

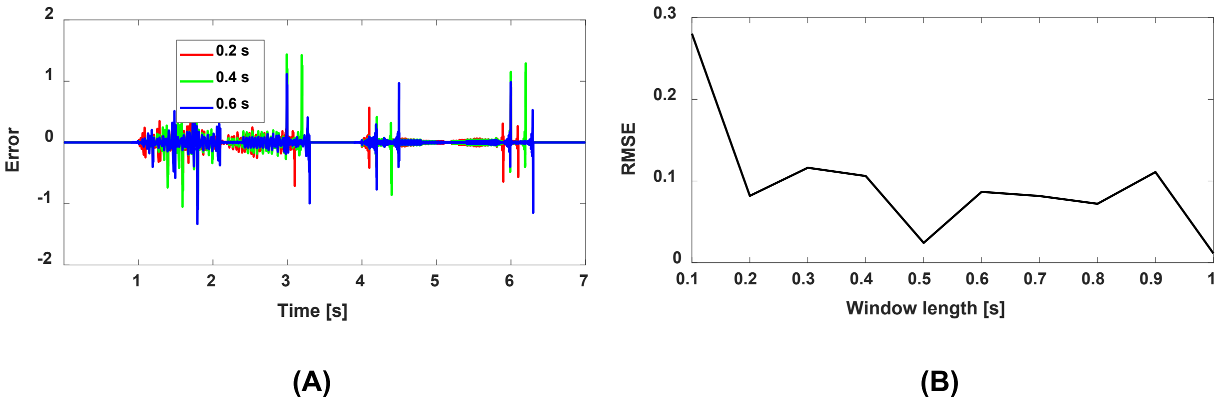

Figure S3. Influence of the window length on filter performance. **(A)** Errors between the filtered signal and the original signal for three representative window length conditions: 0.2 s (red), 0.4 s (green), and 0.6 s s (blue). **(B)** Changes in RMSE based on the window length. RMSE decreases as window length increases.

Window length was changed from 0.1 to 1 second with increments of 0.1 seconds (figure S3). The results showed that RMSE tended to decrease as window length increased (figure S3B). This observation indicates that a longer window length has better accuracy for removing the artifacts. However, a longer window length causes more delay in post-signal processing (i.e., lower time-sensitivity). Thus, window length should be selected depending on which will be focused on accuracy or time sensitivity. However, caution is needed when interpreting the results since simulated artifacts used in this section only addressed stationary noise. In general, real tSCS-contaminated data contains non-stationary noise, such as white noise, and may yield different results.

S1.4 Influence of the length of the overlapping window on filter performance

The size of the overlapping window was changed from 0 to 0.195 seconds with increments of 0.005 seconds. As the size of the overlapping window increased, the filter performance deteriorated (i.e., RMSE increased), as described in figure S4B. Thus, a signal with a minimal size of the overlapping window is desired. However, if real-time processing is considered, a longer size of the overlapping window is desired to increase signal updating speed of post-processing. Thus, the size of the overlapping window should be selected depending on which will be focused on: performance or signal updating speed.

S1.5 Influence of time length of artifact-dominant signals on filter performance

The time length of artifact-dominant signals for filter training (i.e., time length for training) was changed from 0.25 to 1 second with increments of 0.25 seconds. While the current study used 3 seconds of the time length for training, we considered this shorter time length for training to examine the minimum value of the parameter necessary to properly remove the artifacts.

When the time length was set as 0.25 seconds, the abnormal error was observed, as described in figure S5A. This may be induced by artifact components generated from the low frequency condition (4 Hz,

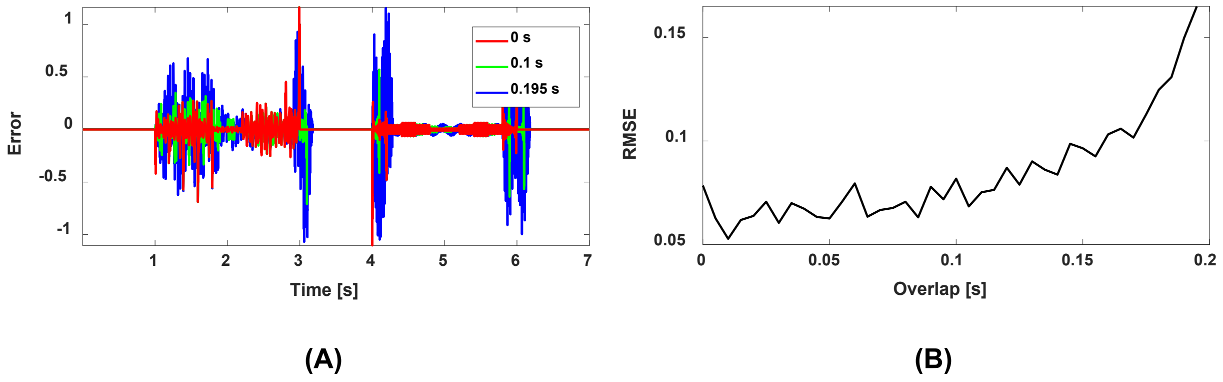

Figure S4. Influence of the size of the overlapping window on filter performance. **(A)** Errors between the filtered signal and the original signal at each time point for three representative overlapping window conditions: $0\,\mathrm{s}$ (red), $0.1\,\mathrm{s}$ (green), and $0.195\,\mathrm{s}$ (blue). **(B)** Changes in RMSE values based on the overlapping window.

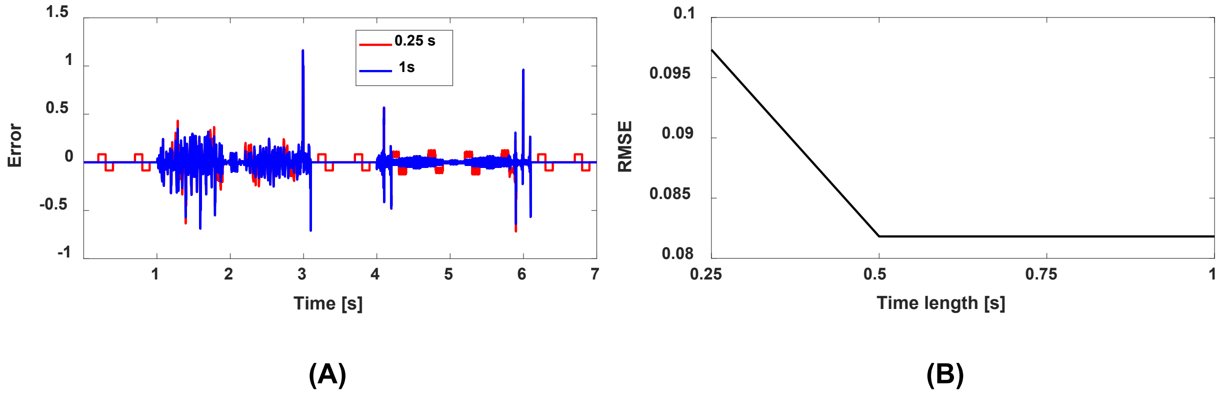

Figure S5. Influence of the time length on filter performance. **(A)** Errors between the filtered signal and the original signal at each time point for two representative time length conditions: $0.25\,\mathrm{s}$ (red), and $1\,\mathrm{s}$ (blue). A time length of $0.25\,\mathrm{s}$ was not sufficient to identify low-frequency artifacts. **(B)** Changes in RMSE values are based on the time length.

$f(4, 1, 0, 0, 7, 7)$). The RMSE gradually reduced when time length ranged between 0.25 to 0.5 seconds and became constant when time length was over 0.5 seconds (figure S5B). This result indicated that 0.5 seconds is the minimum time length for training to cover the low frequency artifact.

Overall, the time length for training should be long enough to cover a wide bandwidth of artifacts. In a case of transcutaneous spinal stimulation (tSCS), the stimulation is typically set to a relatively higher frequency (30-$100\,\mathrm{Hz}$). Therefore, two to three seconds may be sufficient to train all expected artifacts from tSCS.

S2 RATIO BETWEEN PEAK-TO-PEAK SIGNAL AMPLITUDES RECORDED DURING STANDING VS. WALKING

The ACSR filter was designed to identify the frequency distribution of artifacts (i.e., artifact parameters) from sEMG signals that were recorded during a standing posture with the presence of the artifact sources. This approach has a risk for eliminating physiological sEMG signals activated to maintain a standing posture. However, we assumed that the amount of volitional muscle activation necessary for maintaining

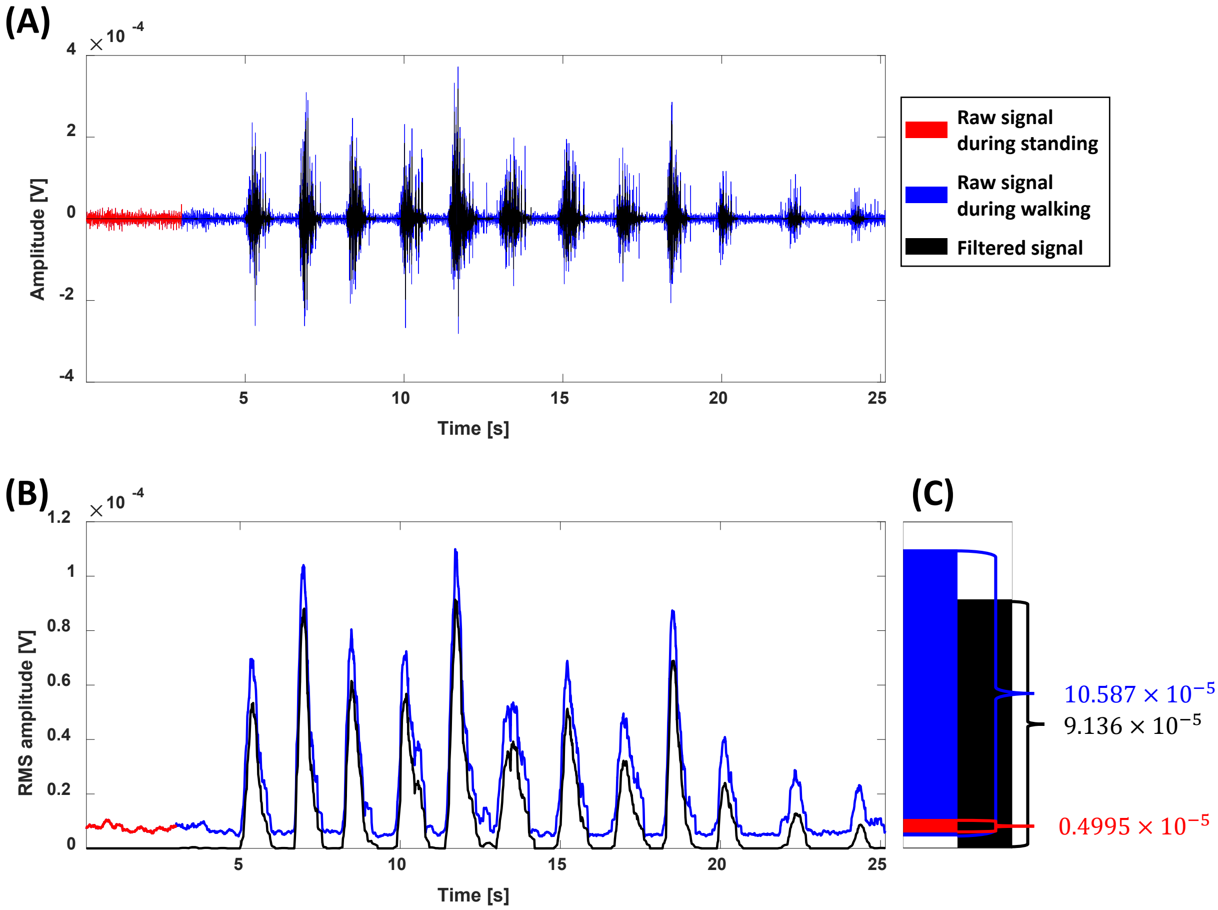

Figure S6. Comparison between the signals recorded during standing vs. walking of an exemplary signal (subject 6, MG muscle, no tSCS condition) **(A)** raw signals during standing (red) and walking (blue) and filtered signals (black), **(B)** RMS enveloped signals (red: standing portion, blue: walking portion, black: filtered signal), **(C)** peak-to-peak signal amplitudes of each portion of RMS enveloped signals (red box: amplitude during standing, blue box: amplitude during walking, black box: amplitude of filtered signal)

standing posture is minor when compared to that during walking. In order to examine this assumption, we systematically computed the ratio between signal amplitudes recorded during standing vs. walking with no stimulation condition of all 6 participants for rectus femoris (RF), vastus lateralis (VL) and medial gastrocnemius (MG), the known muscles activated to sustain the body and maintain balance.

Specifically, the ratio between peak-to-peak amplitudes of the RMS enveloped signals recorded during standing and walking was computed. As an exemplary signals of subject 6's MG muscle recorded during walking without tSCS, is shown in Figure S6A (raw signal: blue line, filtered signal: black line). Figure S6B shows RMS enveloped signals of each standing (red line) and walking portion (blue line), as well as that of filtered signals (black line). Figure S6C visualized peak-to-peak signal amplitudes of each portion of RMS enveloped signals (red box: amplitude during standing, blue box: amplitude during walking, black box: amplitude of filtered signal). As shown in Figure S6C, the amplitude of the signal during standing (red box, 0.4995×10-5[V]) was considerably smaller compared to that during walking (blue box, 10.587×10-5[V]). The ratio between these two amplitudes was 4.718% (=100%×0.4995/10.587). The result indicates that signal amplitude recorded during standing posture is negligibly smaller (less than 5%) compared to that during walking.

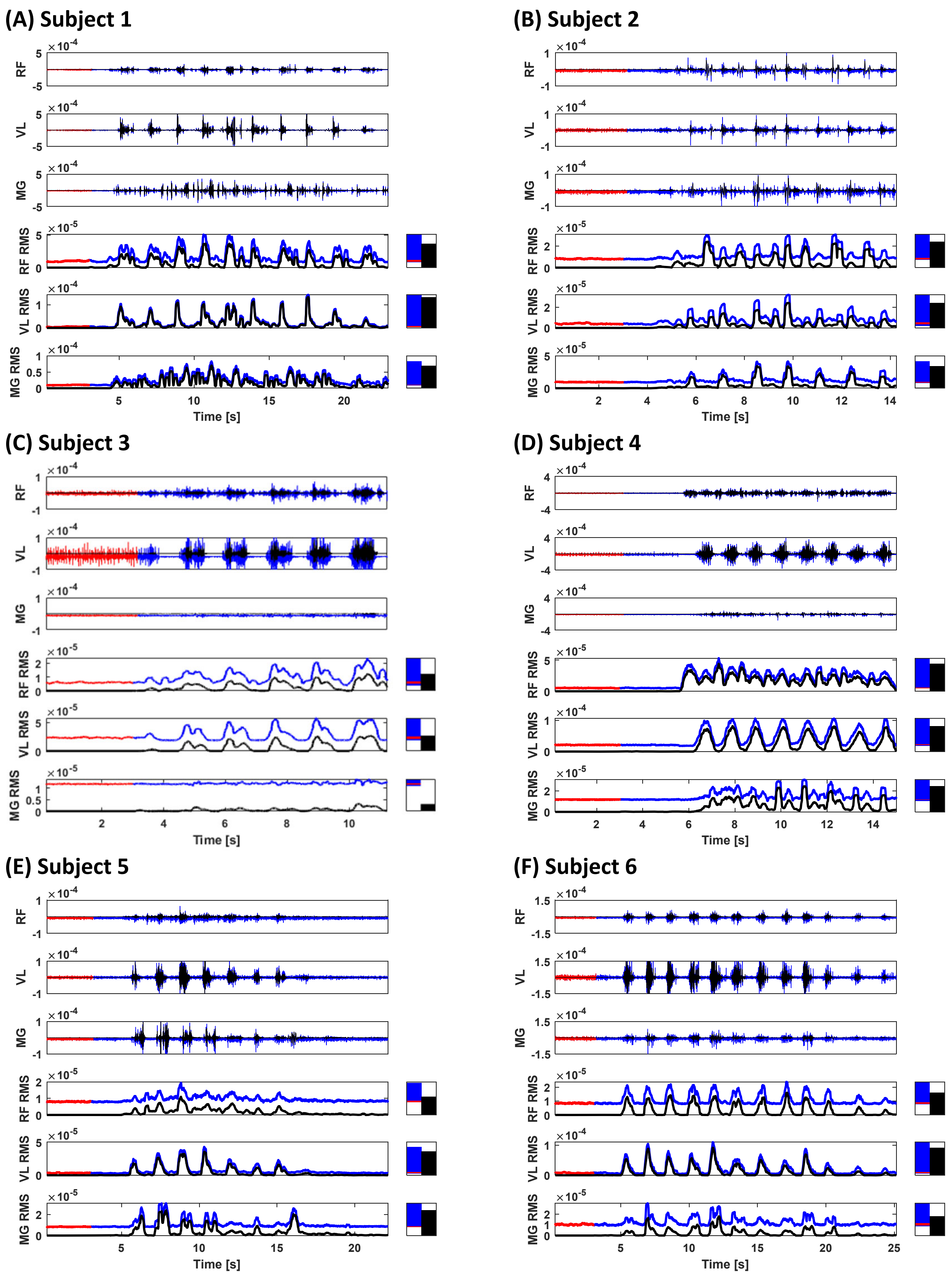

Figure S7. Comparison between the signals recorded during standing vs. walking of all 6 participants' rectus femoris (RF), vastus lateralis (VL) and medial gastrocnemius (MG) muscles recorded with no tSCS. **(A-F)** Subject 1 to 6 participants' data. Each graph shows raw and RMS enveloped signals of each muscle. Red line: standing portion of the unfiltered signal, blue line: walking portion of the unfiltered signal, black line: filtered signal. The box graph shown on the right side represents peak-to-peak amplitudes of each RMS enveloped signal.

		Sub #						AVG
		1	2	3	4	5	6	
Ratio (%)	RF	6.883	6.225	8.346	2.848	8.773	7.885	**6.827**
	VL	3.430	7.076	11.617	3.368	4.401	4.718	**5.768**
	MG	2.963	3.769	19.889	3.176	3.087	11.753	**7.439**
**AVG**		**4.426**	**5.690**	**13.284**	**3.130**	**5.420**	**8.119**	**6.678**

Table S2. Ratio between peak-to-peak amplitudes of RMS enveloped signals recorded during standing vs. walking.

The same method was applied to compute a ratio of signal amplitudes during standing vs. walking for all 6 participants for RF, VL and MG muscles (Table S2 & Figure S7). The results showed that the average of these ratios was 6.678%.

S3 ARTIFACT TO SIGNAL RATIO IN REAL TSCS CONTAMINATED DATA

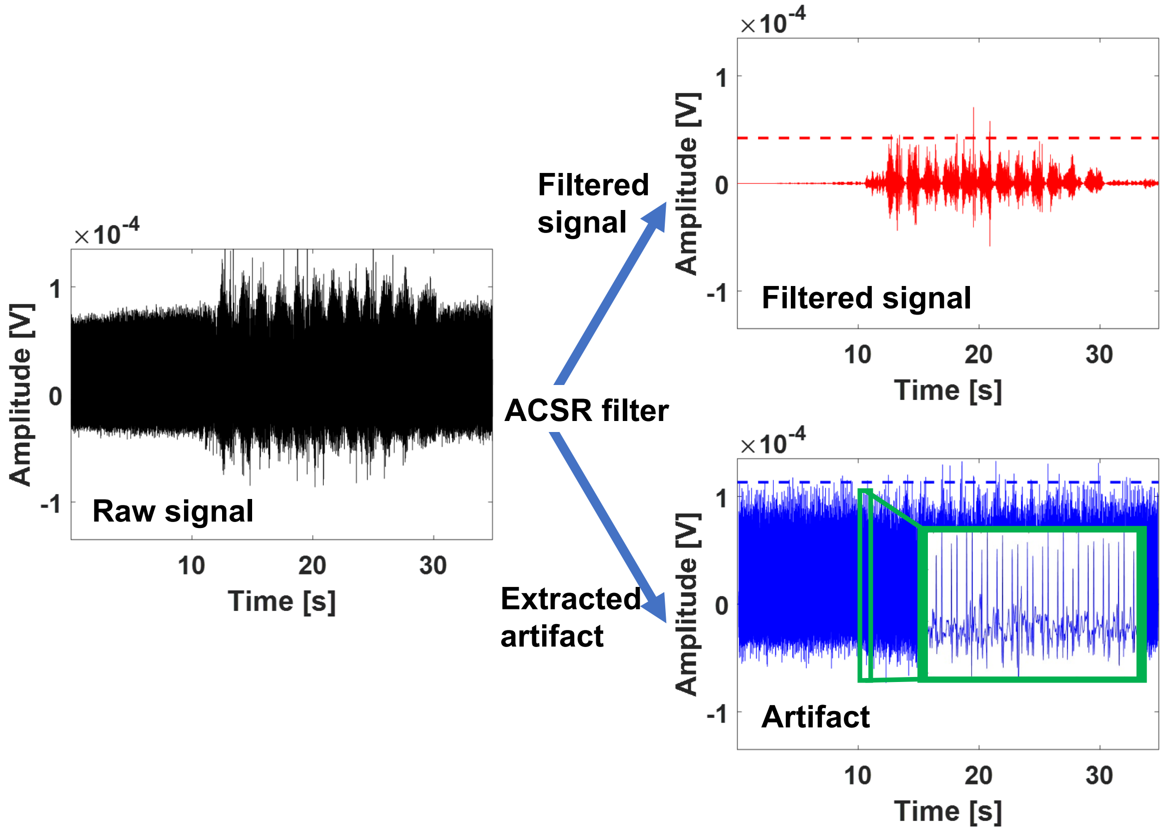

Figure S8. Real tSCS contaminated signal measured from RF of subject 2. The sEMG signal can be decomposed into two signals: the filtered signal and the extracted artifact. Dashed lines of each decomposed signal indicate the power of each signal. The artifact to signal ratio of this signal is 2.682.

Figure S8 describes the sEMG signal measured when tSCS was applied. After identification of artifact parameters, the sEMG signal was decomposed into the filtered signal and the extracted artifact (figure S8, right figures). The artifact signals were extracted from the raw signal from the equations (3), (4), and

following equation:

$$\begin{aligned}|z^n| &= Y^n_{\text{artifact}}\\ \angle z^n &= \angle y^n, \quad n = 1, \cdots, N\\ Z &= \text{ifft}(z)\end{aligned} \tag{S6}$$

where z and Z denote artifact signals in frequency domain and time domain, respectively.

The power of the filtered signal and the extracted artifact were represented by the average of the top 100 signal amplitudes for each signal (dashed lines). In this exemplary signal, the artifact to signal ratio of 2.682 was obtained.
